# Key components and critical factors for developing a telehealth business framework: a qualitative study

**DOI:** 10.1186/s12911-021-01707-3

**Published:** 2021-12-04

**Authors:** Farnia Velayati, Haleh Ayatollahi, Morteza Hemmat, Reza Dehghan

**Affiliations:** 1 Department of Health Information Management, School of Health Management and Information Sciences, Iran University of Medical Sciences, Tehran, Iran; 2 Health Management and Economics Research Center, Heath Management Research Institute, Iran University of Medical Sciences, Tehran, Iran; 3 Department of Health Information Technology, Saveh University of Medical Sciences, Saveh, Iran; 4 Department of Health Entrepreneurship, Virtual University of Medical Sciences, Tehran, Iran

**Keywords:** Small business, Commerce, Medical informatics, Telemedicine

## Abstract

**Background:**

Telehealth technology and related products can help to solve the problems associated with providing health care services and equal distribution of resources. However, in order to run a telehealth business successfully, key components and critical factors need to be taken into account. A telehealth business framework can provide a rich understanding of these components and factors. Therefore, the present study aimed to identify the key components and critical factors for developing a telehealth business framework from the experts’ perspectives.

**Methods:**

The present qualitative study was conducted in 2020. The participants were 22 experts in the fields of medical informatics, health information management, telemedicine, telehealth, health entrepreneurship, health insurance, and digital health start-ups. In depth semi-structured interviews were conducted to collect data, and the data were analyzed using framework analysis.

**Results:**

Four main themes derived from data analysis. The themes included key components for developing a telehealth business framework, success factors, challenges, and barriers of a telehealth business. Overall, the results indicated that the key components in a telehealth business framework included created value, key resources, key activities, key partners, licenses and permissions, product pricing, revenue, marketing, supporting services, and getting feedback from customers. Although receiving support from different individuals and organizations as well as economic benefits of telehealth services may consider as success factors, there are a number of challenges and barriers which should be taken into account. Otherwise, these factors may hinder the success of a telehealth business.

**Conclusions:**

The development of a telehealth business framework might be an important step towards developing a more complete business plan, facilitating the commercialization of telehealth products, and providing a solution for product sustainability in a competitive market. In the current study, the key components and critical factors for developing a telehealth business framework were identified; however, further research is needed to explore how these components and factors can be helpful in developing business plans and running a successful telehealth business.

## Introduction

It is more than a decade that the advancement of Information and Communication Technologies (ICT) has been used to promote the quality and speed of health care services in the form of telehealth and other health care technologies. These technologies can help to solve the problems associated with health care services, equal distribution of resources, and health care costs [1, 2]. Telehealth technology supports the provision of a variety of health care services, such as patient care, tele-education, and tele-consultation, and includes a set of complex technologies, organizational models, clinical services, and evaluation tools. Despite the potentials of this technology to increase the quality of, and access to health care services, its successful implementation and commercialization have always faced several challenges [3, 4]. Although many new health technologies have been accepted clinically and technically, a small number of telehealth innovations has been widely implemented, probably due to not using profitable and sustainable business models (BM) or business plans (BP) [5, 6].

It should be noted that having advanced technology does not guarantee success in the market, and new technologies need to be supported by appropriate business models to survive in the market and make successful commercial deals [7, 8]. Business models play important roles in the implementation of technologies and related products and are used as a basis for interaction with stakeholders [9]. These models provide a path through which innovation, technology, and knowledge are joined together to make the most of the tangible and intangible assets [10]. Although, a variety of business models and plans are used in telehealth industry [11], it seems that providing a telehealth business framework with well-defined components can help the business managers to focus on the most important issues and increase the chance of success. Such a framework is based on understanding the context of telehealth in a broader picture and together with existing challenges and opportunities help to focus on the major aspects of successful telehealth business. It is notable that while a model usually shows a simplification of a phenomenon or a specific aspect of it, a framework usually represents a structure or overview of various descriptive categories, e.g. concepts, constructs or variables, and the relations between them. [12] According to the literature, so far, few studies have been conducted to introduce a telehealth business framework. For example, the CompBizMod framework included four dimensions which were value proposition, value co-creation, value transfer, and value capture [5]. Using this framework, service providers can evaluate their business models and consider components that lead to competitive advantages [5]. However, this framework focused on the value and had few components.

Dargahi et al. introduced a reference business model with three layers; namely, the infrastructure layer, the interface layer, and the business layer, so that it could be used in the development of evaluation criteria for e-health and mobile health technologies at the national and regional levels [13]. However, using such broad components might be difficult for people working in the field of business. In another study, Hasani et al. proposed 24 different factors in six components, including infrastructure management, strategies, financial issues, bidding, environmental management, and technology as a framework for designing and developing an e-health business model [14]. In this framework, general components of e-health business were considered, and the current telehealth business models were not taken into account. It seems that in the previous studies, a review of the general business models was the main part of the research to create the framework and a limited number of components were extracted based on these models. However, in the current study, the aim was to identify the key components and critical factors for developing a telehealth business framework from the experts’ perspectives. This approach helped to gain a deeper understanding of the concepts that should be considered for developing a telehealth business framework.

## Methods

The present qualitative study was conducted in 2020. Before conducting the research, it was reviewed and approved by the review board and ethics committee of Iran University of Medical Sciences (IR.IUMS.REC.1397.1328). In this research, all methods were carried out in accordance with relevant guidelines and regulations of the ethics committee.

### Research participants

Purposive sampling method was used to select the participants of the study [15]. The eligible participants included experts in the fields of health information management, medical informatics, telemedicine and telehealth who had a PhD degree and at least three years of work experience in the fields of telemedicine and telehealth. In addition, key individuals in health entrepreneurship, health insurance, and digital health start-ups who had at least three years of work experience in the field of telehealth business were invited to participate in the interviews. The maximum variations were taken into account when the participants were selected.

### Research instrument

Before conducting the interviews, an interview guide was developed based on the literature review [8, 14, 16]. The content and face validity of the interview guide was approved and validated by three experts in the fields of medical informatics, health information management and entrepreneurship. The interview guide consisted of 19 open-ended questions about the reasons for the success or failure of a telehealth business in a competitive market environment, the business models or plans used to launch a telehealth business, the revenue stream and return on investment, as well as financial resources, necessary licenses, and barriers to a telehealth business (“Appendix”). The interview guide was piloted with three eligible people (who were not the participants of the study) to ensure about the comprehensibility and unambiguity of the questions. Then, minor changes were made and the interview guide was used in the subsequent interviews.

### Data collection

The necessary coordination was performed in advance to interview the experts. One of the researchers (FV) asked the participants to determine a convenient time and place for the interviews. Then, in depth semi-structured interviews were conducted either in the participant’s work place or via video-call due to the spread of COVID_19. Prior to the interviews, the participants were provided with adequate information about the research aim and objectives and were asked to sign a written informed consent. In total, 22 eligible people, who agreed with taking part in the study, were interviewed. The interviews were recorded digitally with the permission of the interviewees and field notes were taken whenever it was necessary. Field notes help to keep a record of the non-verbal behaviors of the interviewees, and can be used when the interviewee does not agree with recoding his/her voice [17]. Interviews continued until data saturation was reached.

### Data analysis

Before data analysis, all interviews were transcribed verbatim. The transcripts were studied several times by one of the researchers (FV) to be familiarized with the scope and variety of key topics. The open, axial, and selective coding were used in data analysis process. Then, the main themes, subthemes and categories were identified and the final interpretation was presented. The coded results and final report were reviewed by other three researchers independently to make sure that there was no misunderstanding or misinterpretation. The data were analyzed deductively using framework analysis [18, 19]. The framework analysis is a systematic and flexible approach for qualitative data analysis. This method includes a hierarchical approach to classify and organize data and has been used in research on health services due to the clarity and transparency of the analysis process [19]. In addition, to facilitate data analysis, MAXQDA software version 12 was used [20, 21]. Finally, a summary of the findings was sent to the interviewees to check the credibility of the results, and all of the participants approved the content.

## Results

In the present study, 45 eligible people were invited to participate in the interviews, and 22 of them agreed to take part. Table 1 presents the participants' characteristics.

**Table 1:** Participants’ characteristics

Variable		Frequency (percentage)
Sex	Male	18 (81.8%)
	Female	4 (18.2%)
Age (years)	30–40	4 (18.2%)
	41–50	15 (68.2%)
	51–60	3 (13.6%)
Education	Ph.D	19 (86.4%)
	M.Sc	3 (13.6%)
Job	Faculty member (assistant professor/ associate professor/ professor)	16 (72.8%)
	Others (Start-up managers, health insurance consultants and health entrepreneurs)	6 (27.2%)
Work experience (years)	< 15	7 (31.8%)
	> 15	15 (68.2%)

It is notable that to respect the confidentiality issues, we de-identified the interviewees’ personal information and used the letter "P" which indicates a participating interviewee and the number following that indicates the specific interviewee providing the quote out of the 22 interviewed.

The research results included four themes, 16 subthemes, and 48 categories as presented in Table 2.

**Table 2:** Themes, subthemes and categories

Themes	Subthemes	Categories
Key components of a telehealth business framework	Created value	Financial value
		Non-financial value
	Key activities	Multi-stage assessment of the product
		Research and analysis
		Business counselling and mentorship
		Effective communication with stakeholders
	Key resources	Human resources
		Physical resources
		Financial resources
	Key partners	Government companies
		Private companies
	Licenses and permissions	Security-based licenses
		General and optional licenses
	Product pricing	Pricing by product manufacturer
		Pricing by an independent organization
	Product revenue	Selling products and data
		Payment by clients
	Product marketing	Traditional marketing
		Virtual marketing
	Supporting services	Supporting manufacturers
		Supporting users
	Getting feedback	Electronically
		In-person
		Research-based
Success factors for a telehealth business	Technical and non-technical support for production	Demand-based production
		Government support
		Healthcare professionals support
		Technical support
	Economic benefits	Accessibility in remote areas
		Having a competitive advantage
Challenges of a telehealth business	Challenges of starting a telehealth business	Time lag between the idea generation and manufacturing
		Ambiguous intellectual property rights
		Lack of trust in business idea centres
		Inadequate support from relevant authorities
		Weakness in teamwork
		Multiplicity of partners
		Lengthy processes
	Product-related challenges	Lack of insurance support services
		Lack of product assessment
		Lack of appropriate reimbursement and tariffs
		Lack of appropriate contracts
		Lack of a business plan
		Lack of a real market
		Heath care professionals’ reluctance to use the product
Barriers of a telehealth business	Legal barriers	Lack of relevant standards and licenses
		Lack of supporting policies and laws
	Financial barriers	Limited financial resources
		Inflation

### Theme 1: Key components of a telehealth business framework

From the interviewee's point of view, the key components of a telehealth business framework included the created value, key activities, key resources, key partners, licenses and permissions, product pricing, product revenue, product marketing, supporting services, and getting feedback. The created value of telehealth products included financial (e.g., saving costs) and non-financial (e.g., improving quality of care) values that directly and indirectly can generate revenue.

Regarding the key activities, most of the interviewees mentioned that multi-stage assessments of the product, conducting research and analysis such as feasibility analysis, risk assessment, market analysis, cost–benefit analysis, business counseling, mentorship, and effective communication with stakeholders are necessary. In this regard, an interviewee pointed out:

…Most of our work does not reach commercialization because we produce a product before we conduct the feasibility study, risk assessment, and market analysis for the products of knowledge-based companies, and we usually fail in commercialization because we do not have the necessary analysis… (P 18).

Key resources included human, physical, and financial resources. Most of the interviewees considered human resources as telehealth staff, experts, and those who are working for marketing, sales, and commercialization. The physical resources included equipment needed to produce telehealth products which could be used either for different products or for a specific telehealth product. From the interviewees' points of view, financial resources were also important in the commercialization and success of the telehealth products and their long-term sustainability in the health market. Financial resources included financial support from the government sector, the private sector, donors, and those interested in investment.

In terms of the key partners, some interviewees noted that the commercialization process is much easier without having a business partner. However, if there is a need for a business partner for the success of the business, the business partner must be selected from government or private companies by signing specific and clear contracts. An interviewee, who was the manager of a health insurance organization, noted that:

…Depending on the type of product, all stakeholders must be considered, and a suitable business partner should be selected from them… (P 16).

Regarding licenses and permissions, the results indicated that the telehealth market needs specific licenses so that people can trust telehealth products. The interviewees explained different types of licenses such as security-based, general and optional ones. The findings also showed that the importance of telehealth has increased after the COVID_19 pandemic. However, despite the increased use of telehealth technologies, licenses are still important issues and need further attentions (P 2).

Regarding telehealth product pricing, the interviewees pointed out that tangible and intangible costs of production as well as prices of other competitors' products in the market should be taken into account in pricing strategy. In this case, the product manufacturer can gain the maximum profit. As an interviewee noted:

…Prices should be set by product manufacturers based on a series of economic models and service values, and pricing should be based on different strategies for different stakeholders… (P 21).

Some of the interviewees believed that telehealth product pricing should be performed by an independent organization. This organization can play a supervisory role, and all manufacturers of telehealth technology need to follow rules and pricing strategies.

Product revenue was another key component in a telehealth business framework which should be determined based on the product cost and purpose, the service delivery features, and types of customers. The interviewees believed that they could generate revenue through selling products and data to other researchers and research centers, and receive payments periodically.

Product marketing included traditional and virtual marketing. Many interviewees believed that telehealth products can be advertized in different conferences and scientific associations. Several interviewees noted that virtual marketing is easier and more cost-effective than other advertizing methods. Moreover, it provides easy access to all target groups.

From the interviewees' points of view, providing supporting services for manufacturers and users of telehealth technologies are of high importance for a successful business. Supporting users can be based on getting feedback from them. The feedback can be obtained electronically, in-person, or research-based.

### Theme 2: Success factors for a telehealth business

The interviewees also mentioned a number of success factors for a telehealth business. These factors included technical and non-technical support for production and economic benefits of a telehealth product. From the interviewees' points of view, factors such as demand-based production, government support, healthcare professionals' support, and technical support are necessary for a successful telehealth business. As an interview indicated sometimes government support can be demonstrated as mandatory use of a product:

…Sometimes, the telehealth use should be mandatory as a premier solution such as COVID-19 situation, and thus the use of a telehealth product should become mandatory from the government… (P 14).

Some of the interviewees also believed that the economic benefits of technologies, such as being available in remote areas, saving travel cost and time, facilitating access to health care services and having competitive advantages like increasing market share of the potential telehealth users can help to gain success in a telehealth business. In this regard, an interviewee said:

…A telehealth product must have economic justification and competitive advantages so that the customers can pay for the product… (P 12).

### Theme 3: Challenges of a telehealth business

Telehealth business challenges included challenges of starting a telehealth business and product-related challenges. Most of the interviewees believed that there are a number of challenges at the very beginning of a telehealth business. These challenges include the time lag between the idea generation and manufacturing, ambiguous intellectual property rights, lack of trust in business idea centers, inadequate support from relevant authorities, weakness in teamwork, multiplicity of partners, and lengthy processes for starting a new telehealth business. In this regard, an interviewee said:

…We worked with three large organizations to produce products, and the negotiations and administrative processes between the organizations took a long time. At the end, very complicated conditions arose in terms of intellectual property rights, and the work did not come to fruition… (P 1).

Furthermore, there are also product-related challenges which include the lack of insurance support services, lack of product assessment, lack of appropriate reimbursement and tariffs by legal authorities, lack of appropriate contracts, lack of business planning, lack of a real market, and health care professionals' reluctance to use the products. The interviewees believed that legal and insurance processes were needed for the success of a telehealth product. However, most of the telehealth interventions have a preventive or supportive nature, and there is often no preventive or supportive view in the health insurance companies to cover the product costs. Moreover, legal and insurance challenges cause the clinical sector to move less towards using the technology or to pay less out-of-pocket for using it. Some interviewees noted that although their products met the technical principles and users’ requirements, they faced problems due to the lack of a proper business plan to make revenue. The contracts also need to be supervised by highly qualified professionals to consider all legal aspects.

### Theme 4: Barriers of a telehealth business

The results indicated that the barriers ahead of a telehealth business may prevent product commercialization. These barriers included legal and financial barriers. The lack of relevant standards and licenses, and the lack of related policies and laws were among the legal barriers mentioned by the interviewees. In this regard, an interviewee said:

…Stability in policy-making and regulations paves the way for commercialization… (P 7).

Financial barriers included limited financial resources and inflation. The participants believed that the research budgets were limited, and they had to pay high costs for equipment and conducting necessary studies. The interviewees also believed that the prices of equipment would be different at the end of the production compared to the prices at the start of a telehealth business. This is mainly due to inflation, which affects product pricing or the manufacturer's profit.

## Discussion

Although the use of telehealth technology has largely increased around the world, there are still a number of certain issues, such as proposed values, budget allocation, legal supervision, licenses, resources, technical and business issues which need to be taken into account [22]. Van Limburg et al. explained that the sustainability of telehealth technology is low mainly due to some challenges such as the lack of technical infrastructure, limited financial resources, inappropriate scalability, and uncertainty about the effectiveness of the technology. However, these issues can be taken into account before running a telehealth business by creating an appropriate business plan [9]. To develop an effective business plan, a telehealth business framework can work as a reference model to address key components and critical factors for a successful telehealth business. Therefore, the present study aimed to identify the key components and critical factors for developing a telehealth business framework from the experts’ perspectives.

The results indicated that the key components of the telehealth business framework were as follows: created value, key resources, key activities, key partners, licenses and permissions, product pricing, product revenue, product marketing, supporting services, and getting feedback from customers. In fact, paying attention to these components is important to facilitate product commercialization before running a telehealth business. It should be noted that these components are related to each other, and any changes in one component may influence other components. As Chen et al. highlighted, a deeper understanding of the nature of each component is necessary to gain success in a telehealth business [23]. For example, created value can be different in each telehealth technology and can directly or indirectly affect users’ attitudes towards using the technology. Similarly, Acheampong and Vimarlund indicated that the created value in telemedicine was diverse and often referred to improving the quality of care, accessibility of health care services, and efficiency of care [24]. Other studies also emphasized the value proposition and diversity of this component in the fields of mobile health and telehealth in different country [23, 25].

Similar to the created value, key activities can be different in various telehealth businesses and include a range of activities that a business needs to undertake to deliver the value to the customers. The study conducted by Chen et al. showed that key activities might be related to the product design or a set of research activities before production [23].

Among key resources, financial resources are of high importance in commercialization, the success of a telehealth business, and the long-term sustainability of a telehealth product in the health market. In a similar study conducted by Arkwright et al., the results showed that different financial resources, such as fee-for-service (FFS) payments, value-based payments, periodic payments of clients, and external funding had a positive impact on the success of a telehealth business [26]. However, Effertz et al. found that there are five general approaches to sustaining a telehealth center. These are grants, telehealth network membership fees, income from providing clinical services, per encounter charges, and operating as a cost center and most centers use more than one approach [27].

According to the results, key partners in a telehealth business should be identified, too. The success of a telehealth business depends more on the collaboration between partners and stakeholders rather than the technology. The success of such collaborations and partnerships also depends on the ability of the public sector and the willingness of the private sector to participate in a business [28].

Regarding licenses and permissions, while some of them might be general and optional, the security-based ones are of high importance in a telehealth business, and the telehealth products should be eligible to obtain them. Similarly, the results of the research conducted by Wijesooriya showed that telehealth technology licenses might be different across a country or around the world and obtaining them is a main challenge of telehealth businesses [29].

Product pricing and revenue were other key components in a telehealth business framework. It seems that the price of telehealth products is one of the main challenges ahead of the widespread deployment of telehealth services. According to Pereira, the pricing mechanism should be transparent and follow an appropriate pricing method, such as pay-as-you-go, subscription, the buffet model, or micropayments. In addition, pricing can be considered as a component of the revenue model in which other aspects, such as partner revenue sharing, product cost structure, and potential volume of the product should be taken into account [6].

In terms of product marketing, the findings indicated that the telehealth product marketing methods might be different from other types of marketing mainly due to the wide range of technologies, customers, stakeholders, and services in a telehealth business. Providing adequate supporting services for manufacturers and customers can facilitate marketing and increase revenue. It is also essential to identify the end-users and customers before running a telehealth business and determine their requirements [24].

As customers’ opinions are important for sustainability of a business, their feedback should be investigated regularly, as they may influence the success or failure of a telehealth business. In fact, due to the wide range of customers, many contextual and cultural factors must be considered in a telehealth business [23]. Moreover, to evaluate the quality and impact of telehealth products and services, some of the highest-priority dimentions, such as access to care, financial impact, patient’s and provider’s experience and effectiveness need to be measured to inform future telehealth quality improvement efforts [30].

Apart from the key components, a number of critical factors should be taken into account in developing of a telehealth business framework. The results showed that success factors, challenges and barriers a head of a telehealth business may influence making decision about running a telehealth business. Regarding success factors, the results showed that technical and non-technical support for a product and economic benefits are of great importance for a telehealth business. Similarly, some researchers found that paying attention to the technological, organizational, and managerial issues can help to reach success in a large scale implementation [31, 32]. It seems that making an agreement between health care organizations and a telehealth business and involving health care professionals in the process of marketing can also affect the success of a telehealth product [32].

As the results showed, there is no guarantee of success in starting a telehealth business due to some challenges and barriers. The results of other studies have also indicated that telehealth technologies face different challenges and need a legal framework for licenses, compensation, responsibility, data sharing, and data protection [30, 33, 34]. Moreover, the nature of technology commercialization is complex and challenging, because customers’ requirements change quickly, and the technology life cycle is short in the modern technology environment [35].

The results of the present study also indicated that legal and financial barriers to the telehealth business may prevent running or continuation of a successful business or hinder the commercialization of telehealth products. Therefore, legal actions need to be taken in some important areas like reimbursement, privacy/security, liability, and issuing licenses [36]. In terms of financial barriers, allocating more resources are necessary, as most of the telehealth products need long-term financial support [25].

Overall, the current study contributed to introduce key components and critical factors in developing a telehealth business framework. Such a framework can play an important role in reducing risks and costs and can increase the likelihood of success in a telehealth business [22]. Moreover, it can facilitate developing a business plan, which, in turn, is a strategic tool for business success [37, 38].

### Limitations

The current research had some limitations. Although the number of interviewees was limited, we tried to interview the most informed people who had adequate knowledge about both telehealth and business. Moreover, as the main aim of the research was to identify key components and critical factors for a telehealth business framework, we sought to present it as general as possible rather than focusing on a specific telehealth business. Obviously, when a business plan is created for a telehealth business, many other issues and influencing factors might be experienced in practice, which are considered in our study. Therefore, more research is needed to address the limitations of the current study and develop a more comprehensive framework to be used in practice effectively.

## Conclusion

The present study aimed to identify the key components and critical factors for developing a telehealth business framework from the experts’ perspectives. The key components were created value, key activities, key resources, key partners, licenses and permissions, product pricing, revenue, marketing, supporting services, and getting feedback. The success factors, challenges and barriers of a telehealth business were the critical factors which could influence the success or failure of a telehealth business and should be taken into account along with the key components when developing a business plan. A telehealth business framework can be used as a reference model to develop telehealth business models and plans. It also shows necessary aspects of a successful telehealth business and is a step towards increasing revenue and profit. However, further research is recommended to validate the key components by other experts in the related fields and to examine the application of the framework in practice.

## Data Availability

The data used and analyzed during the current study are available from the corresponding author on reasonable request.

## Appendix: Interview guide

**Table:**

1. Could you please explain your current and previous experiences in the field of telehealth?
2. Please talk about your projects or ideas which have been turned into a telehealth business
3. Please talk about your projects or ideas which have not been turned into a telehealth business. Why?
4. Could you please explain the business models and business plans that you have ever used? Why did you use them?
5. Can you talk about the reasons for success or failure of a telehealth business?
6. Can you talk about the reasons for the success or failure of a telehealth product or service in a competitive market environment?
7. Why should you use business models or business plans to launch a telehealth business?
8. How can you introduce a telehealth product or service to the market?
9. How can you encourage the customers to use a telehealth product or service?
10. How do you plan for the revenue stream of a telehealth product or service and return on investment?
11. How does a telehealth product or service create value for the costumers?
12. Please explain the key resources for the production of a telehealth product
13. Please explain the key activities for the production of a telehealth product
14. How do you choose the key partners in a telehealth business?
15. How do you provide financial resources, cost structure, and product pricing?
16. How do you receive licenses for a telehealth product or service?
17. How do you use support services for a telehealth product?
18. Why and how do you get feedback from the telehealth customers?
19. Can you explain barriers ahead of a telehealth business?

## Abbreviations

ICT: Information and Communications Technologies
BM: Business models
BP: Business plan
FFS: Fee-for-service
